# Longitudinal analysis of cardiac structure and function in incident-automated peritoneal dialysis: comparison between icodextrin solution and glucose-based solution

**DOI:** 10.1186/s12882-018-0912-7

**Published:** 2018-05-08

**Authors:** Jin-Bor Chen, Ben-Chung Cheng, Wen-Hao Liu, Shang-Chih Liao, Mao-Young Morgan Fu, Sin-Hua Moi, Cheng-Hong Yang

**Affiliations:** 1 Division of Nephrology, Department of Internal Medicine, Kaohsiung Chang Gung Memorial Hospital, Chang Gung University College of Medicine, 123 Ta Pei Rd, Niao Song District, Kaohsiung, Taiwan; 2 Division of Cardiology, Department of Internal Medicine, Kaohsiung Chang Gung Memorial Hospital, Chang Gung University College of Medicine, Kaohsiung, Taiwan; 3 Division of Nephrology, Kaohsiung Municipal Feng-Shan Hospital, Kaohsiung, Taiwan; 4 Department of Electronic Engineering, National Kaohsiung University of Applied Sciences, Kaohsiung, Taiwan

**Keywords:** Icodextrin solution, Glucose-based solution, Echocardiogram, Automated peritoneal dialysis

## Abstract

**Background:**

This study aimed to evaluate the longitudinal changes in cardiac structure and function in incident-automated peritoneal dialysis (APD) patients.

**Methods:**

We conducted a 2-year prospective, randomized, open-label, parallel-group study to compare the efficacy of icodextrin solution versus glucose-based solution. Echocardiography was performed at baseline, 1 and 2 years. Echocardiographic parameters over 2 years were evaluated for each group, using the Friedman test. Generalized linear regression analysis was used to test the associations between baseline clinical variables and echocardiographic changes, and a multivariate model was used to analyze cardiac function between the two groups.

**Results:**

A total of 43 APD patients were enrolled in the beginning of this study. Twenty patients in the icodextrin group (ICO) and 18 patients in the glucose group (GLU) completed the study. In left ventricular (LV) systolic function measurements, ejection fraction (EF) increased significantly in the GLU group. Measurements of LV diastolic function and septal early mitral annulus velocity (EMV) increased significantly from baseline to 24-months in the ICO group (5.43–5.51 ms). The GLU group showed a significant decrease in peak early diastolic velocity (EDV) (70.67–68.25 cm/s), but a significant increase in septal EMV (5.94–7.57 ms) from baseline to 24-months. No significant association was found between the baseline clinical variables and echocardiographic changes within 24 months in the generalized linear regression analysis. Multivariate models were used to investigate changes in the four primary endpoints, namely, myocardial performance index (MPI), left ventricular ejection fraction (LVEF), deceleration time (DT), and *E*/*e*′ ratio. These primary endpoints show no significant association with the baseline values in both the ICO and GLU groups.

**Conclusion:**

The present study demonstrates that long-dwell icodextrin solution can maintain reasonable cardiac structure and function in incident-APD patients.

**Trial registration:**

ISRCTN14931270 (retrospectively registered on 23/03/2018).

## Background

Cardiovascular disease has been recognized as the leading cause of mortality in patients with chronic kidney disease (CKD) [1]. Among the clinical manifestations of patients with CKD, heart failure is the most prevalent. Clinical presentation is commonly preceded by structural cardiac disease, evidenced by image examinations [2, 3]. A prior study revealed that CKD patients exhibited increased left ventricular (LV) mass index, left atrial volume index, and diastolic dysfunction, with their status deteriorating with the progression of CKD [4]. Another study investigated cardiac function in pre-dialysis CKD patients, and revealed diastolic dysfunction indicated by an increase in the ratio of mitral inflow velocity and mitral annulus velocity, and left atrial volume indexed for height, prior to dialysis initiation [3]. In addition, a previous study demonstrated that, among prevalent peritoneal dialysis (PD) patients, those without a history of heart failure had an increased ratio of mitral inflow velocity and mitral annulus velocity, and that deceleration time indicated diastolic dysfunction [5]. Thus, evaluation of cardiac structure and function in CKD patients is crucial in long-term CKD management.

Icodextrin (ICO) is a glucose polymer with an average molecular weight of 17,000 Da. It can act as a colloid osmotic agent in PD therapy. ICO solution has provided advantages over glucose-based PD solution for long-dwell exchange with sustained net ultrafiltration to 16 h on continuous ambulatory peritoneal dialysis and APD, with less damage to the peritoneal membrane [6]. In one previous study with high transport diabetic PD patients, the ICO solution demonstrated advantages in peritoneal ultrafiltration and fluid control, and facilitated metabolic control compared with the glucose-based solution in a 12-month study period [7]. Moreover, one study assessed the ultrafiltration effect of ICO solution in the nocturnal schedule of APD, showing that the ICO solution might allow sustained ultrafiltration and sodium removal [8]. Regarding the association between ICO use and echocardiographic parameters in PD patients, a study revealed significantly decreased left ventricular mass and extracellular water over a 4-month observation period [9]. Nevertheless, more detailed echocardiographic parameters are not available in the literature. Thus, a well-designed prospective study is needed to address the effect of ICO solution on cardiac structure and function in PD patients.

We hypothesized that the use of ICO in PD therapy has an advantage in cardiac function via sustained ultrafiltration compared to GLU-based solution. In the present study, we aimed to compare the impact of ICO- and GLU-based solutions on cardiac structure and function in a 2-year longitudinal period in incident-APD patients. A prospective randomized study was conducted in a cohort of APD patients who were undergoing a newly initiated APD therapy with long-dwell exchange in the daytime. A series of echocardiographies were performed to assess cardiac structure and function during the study period.

## Methods

This prospective, randomized, open-level, parallel-group study was started in June 2009 and was completed in May 2015. All of the participants were selected from the PD unit in Kaohsiung Chang Gung Memorial Hospital in Taiwan. The main inclusion criterion was adult incident-PD patients who agreed to receive nocturnal APD regimen with daily dwell. We used a purposive sampling method to enroll study participants in the outpatient department. After the study protocol was explained and informed consent was obtained, we used a computer-generated block randomization method to categorize enrolled participants into two groups. All of the participants underwent nocturnal APD with varying concentrations of glucose-based PD solutions (1.36, 2.27, and 3.86%; Baxter Healthcare SA, Singapore) depending on the prescription from their respective nephrologists. In the ICO group, the participants received long-dwell exchange for 10–12 h with a 7.5% ICO PD solution (Baxter Healthcare SA, Singapore) in the daytime. In the GLU group, the participants received 1 or 2 exchanges with the glucose-based PD solution in the daytime. Cardiac structure and function were examined on echocardiography at baseline and subsequently in 1-year intervals. The study duration was 2 years.

**Fig. 1:**
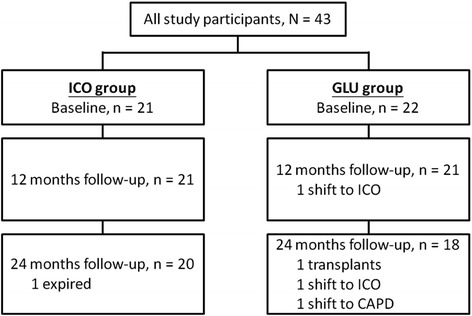
Flowchart of patient selection and follow-up status

The inclusion criteria for the study were age older than 18 years, ability to provide written informed consent, and new incident stage 5 CKD patients who agreed to receive renal replacement therapy with the APD regimen and tolerate a long-dwell time of ≥10 h with 7.5% ICO solution. The exclusion criteria were starch allergy, glycogen storage disease, a life expectancy of < 12 months, serious disease within 30 days before randomization, participation in another interventional study, pregnancy or lactation, or a significant psychiatric disorder that would interfere with their ability to provide informed consent and/or comply with the study procedures. Finally, 43 patients who met the inclusion criteria were enrolled, and 38 patients completed the study (Fig. 1).

During the study period, the patients were requested to visit the PD outpatient clinic at least once every month, and were examined by PD nurses using the PD protocol through telephone contact at least once every week. Data were collected from the PD home record, including body weight, blood pressure level, ultrafiltration (UF), amount, and concentration of the PD solution used. Laboratory data, including hemogram and biochemistry results, were examined at baseline and monthly thereafter. Blood levels of intact parathyroid hormone (iPTH), high-sensitivity C-reactive protein (hsCRP), and glycohemoglobin (HbA1c) were measured at baseline and every 6 months. All blood samples were measured by using commercial kits and an auto-analyzer (Hitachi 7600–210, Hitachi Ltd., Tokyo, Japan). Albumin levels were measured using the bromocresol green method.

Standard peritoneal equilibration test (PET) was performed 1 month after APD commencement. The procedure used a 4-h dwell for a 2.27% glucose-containing PD solution. Twenty-four urine and dialysate samples were collected for measurement of residual glomerular filtration rate, and total weekly urea and creatinine clearance. Thereafter, PET was performed every 6 months within the study period.

Echocardiographic examinations (two-dimensional and M-mode) were performed in all participants by an experienced cardiologist using an ultrasonography system (model 260 SS-A equipped with a 2.5-MHz phased array transducer, Toshiba, Tokyo, Japan). All of the patient examinations were performed in the left lateral recumbent position. All echocardiographic data were evaluated according to the guidelines of the American Society of Echocardiography.

Cardiac performance was evaluated using pulsed Doppler echocardiography, which was conducted at the left ventricular inflow tract under two-dimensional guidance in the apical four-chamber view. The sample volume was positioned at the tips of the mitral valve leaflets and aligned to make the angle between the ultrasound beam and the blood flow vector, as determined based on color flow images, as close to zero as possible. Echocardiographic data were evaluated for cardiac structure (left atrial diameter [LAD], left ventricular end-diastolic dimension [LVEDD], left ventricular end-systolic dimension [LVESD], left ventricular end-diastolic volume [LVEDV], left ventricular end-systolic volume [LVESV], interventricular septal thickness [IVS]), left ventricular systolic function (myocardial performance index [MPI], and left ventricular ejection fraction [LVEF]), and left ventricular diastolic function (deceleration time [DT], peak early diastolic velocity, and septal early mitral annulus velocity). LVEF and MPI were calculated using standard formulas and data obtained from echocardiography. *E*/*e*′ indicated left ventricular diastolic function, and was calculated by dividing the peak early diastolic velocity by the septal early mitral annulus velocity.

After commencement of the study, patient medications, including antihypertensives, were adjusted according to clinical need. PD glucose concentration and fill volume were also adjusted as necessary. The study protocol was approved by the Committee on Human Research at Kaohsiung Chang Gung Memorial Hospital (98-0390B). The study was conducted in accordance with the Declaration of Helsinki.

The baseline characteristics of the participants were summarized and presented as mean ± standard deviation (SD), median and interquartile range, frequency, and percentage. The differences in baseline characteristics between the two groups were estimated by using the *χ*^2^ test and independent two-sample *t* test. Echocardiographic parameters from the baseline to 12 and 24 months of observation were evaluated for each group using the Friedman test. Associations between baseline variables and echocardiographic changes in the study period were analyzed by using a generalized linear regression model. Multivariate linear regression analysis was used to evaluate the 24-month changes in the four primary end points(MPI, LVEF, DT, and *E*/*e*′).

## Results

At the beginning of the study, 43 participants were enrolled - 21 participants in the ICO group, and 22 participants in the GLU group. Twenty participants in the ICO group, and 18 participants in the GLU group completed the study (Fig. 1). Males were predominant in the ICO group; and females were predominant in the GLU group. Diabetic nephropathy was more prevalent in the ICO group. Baseline levels of Hb, Cr, cholesterol, daily ultrafiltration amount and weekly renal Kt/V significantly differed between the two groups (Table 1).

**Table 1:** Baseline laboratory and peritoneal equilibration test data

Variable	Icodextrin (*n* = 21)		Glucose (*n* = 22)		*P*
	Mean	SD	Mean	SD	
Age (years)	44.37	12.59	48.22	12.87	0.327
Sex					< 0.001
Male (n, %)	15	71.43	5	22.73	
Female (n, %)	6	28.57	17	77.27	
DM nephropathy (n, %)	6	28.57	1	4.55	0.046
Antihypertensive (n, %)	16	76.19	14	63.64	0.241
ACEi/ARB (n, %)	15	71.43	10	45.45	0.051
PET					0.077
High (n, %)	5	23.81	0	–	
High average (n, %)	9	42.86	9	40.91	
Low average (n, %)	5	23.81	10	45.45	
Low (n, %)	2	9.52	3	13.64	
SBP (mm Hg)	138.38	20.31	131.41	19.75	0.261
DBP (mm Hg)	85.10	9.86	85.91	12.66	0.816
UF amount^a^(ml/day)	1100	(663–1400)	775	(600–900)	0.021
BMI	23.73	3.26	22.61	4.90	0.387
HbA1C (%)	5.68	1.01	5.47	0.89	0.486
Hb (g/dL)	9.70	1.45	11.13	1.58	0.004
Hct (%)	29.23	5.43	34.05	4.67	0.003
Albumin (g/dL)	3.65	0.39	3.66	0.42	0.954
BUN (mg/dL)	59.33	15.93	59.09	21.03	0.966
Cr (mg/dL)	11.32	2.00	9.55	2.65	0.018
Sugar (mg/dL)^a^	98	(84–106)	94.5	(89–103)	0.455
Calcium (mg/dL)	9.06	1.04	8.83	0.59	0.374
P (mg/dL)	4.97	1.51	4.82	1.17	0.719
Na (meq/L)	132.00	17.34	139.18	3.39	0.064
K (meq/L)	3.94	0.65	3.94	0.43	0.992
i-PTH (pg/mL)^a^	151	(93.7–286)	328.5	(113–602)	0.991
Cholesterol (mg/dL)^a^	166	(130–198)	210.5	(180–241)	0.001
Triglyceride (mg/dL)^a^	135	(78–178)	89	(72–178)	0.371
hsCRP (mg/L)	3.91	4.03	6.04	9.61	0.353
Renal Kt/V weekly	0.45	0.27	0.74	0.41	0.011
Total Kt/V weekly	2.04	0.36	2.18	0.46	0.264
Renal Ccr weekly	22.66	14.33	29.69	18.40	0.171
Total Ccr weekly	62.90	13.95	64.92	16.83	0.671
PGLI	0.31	0.09	0.32	0.16	0.768

*P* value is estimated from the Fisher’s exact, *χ*^2^ or independent two-sample *t*-test

*Abbreviations*: *ACEi* angiotensin converting enzyme inhibitor, *ARB* angiotensin receptor blocker, *SBP* systolic blood pressure, *DBP* diastolic blood pressure, *UF* ultrafiltration, *BMI* body mass index, *hsCRP* high-resolution C-reactive protein, *PGLI* peritoneal glucose loading index

^a^ Data are presented as median (interquartile range)

Compared with the ICO group, the GLU group showed significantly lower baseline LVESD (35.00 mm vs 30.49 mm), and LVESV values (53.48 mm^3^ vs 39.00 mm^3^) (Table 2).

**Table 2:** Baseline echocardiographic parameters

Variable	Icodextrin			Glucose			*P*
	*n*	Mean	SD	*n*	Mean	SD	
Cardiac structure							
LA diameter (mm)	21	34.97	7.51	22	32.25	5.88	0.193
LVEDD (mm)	21	50.80	7.66	22	46.88	6.36	0.075
LVESD (mm)	21	35.00	6.35	22	30.49	6.14	0.023
LVEDV (mm^3^)	21	124.71	42.49	22	103.95	34.34	0.085
LVESV (mm^3^)	21	53.48	21.83	22	39.00	22.22	0.037
IVS (mm)	21	12.65	2.29	22	11.53	1.79	0.082
LV systolic function							
MPI	21	0.37	0.19	22	0.49	0.57	0.367
LVEF (%)	21	57.81	10.03	22	63.40	9.51	0.068
LV diastolic function							
Deceleration time (ms)	21	169.81	73.50	22	213.59	85.21	0.079
Peak early diastolic velocity (cm/s)	20	71.89	34.66	22	70.67	28.75	0.902
Septal EM velocity (ms)	21	5.43	3.00	22	5.94	1.58	0.482
*E*/*e*′	20	11.70	6.36	22	12.38	5.24	0.707
RV function							
TAPSE	21	20.96	3.28	22	21.07	2.64	0.900
PAsP	21	20.65	9.70	22	18.81	4.82	0.409

*P* value was estimated from the independent two-sample *t* test

*Abbreviations*: *LA* left atrium, *LVEDD* left ventricular end-diastolic dimension, *LVESD* left ventricular end-systolic dimension, *LVEDV* left ventricular end-diastolic volume, *LVESV* left ventricular end-systolic volume, *LVEF* left ventricular ejection fraction, *IVS* interventricular septum, *MPI* myocardial performance index, *EM* early mitral annulus, *E*/*e*′ peak early diastolic velocity/septal EM velocity, *RV*right ventricle, *TAPSE* tricuspid annular plane systolic excursion, *PAsP* peak systolic pulmonary pressure

Cardiac structure measurements, including LAD, LVEDD, LVESD, LVEDV, LVESV, and IVS, in both groups were significantly altered from baseline to 24 months. In the ICO group, LAD significantly increased, while LVEDV, LVESV, IVS significantly decreased. In the GLU group, LAD, LVEDD, LVESD, LVEDV, and LVESV all significantly increased. In LV systolic function measurement, only LVEF had a significant increase in the GLU group. In the measurements of LV diastolic function, only septal EMV showed significant increase from baseline to 24 months in the ICO group (5.43–5.51 ms). The GLU group showed a significant decrease in peak EDV (70.67–68.25 cm/s), but a significant increase in septal EMV (5.94–7.57 ms) from baseline to 24 months (Table 3).

**Table 3:** The series of echocardiographic parameters among the icodextrin and glucose users

	Cardiac structure					
	LAD (mm)	LVEDD (mm)	LVESD (mm)	LVEDV (mm^3^)	LVESV (mm^3^)	IVS (mm)
Icodextrin						
Baseline (*n* = 21)	34.97 ± 7.51	50.80 ± 7.66	35.00 ± 6.35	124.71 ± 42.49	53.48 ± 21.83	12.65 ± 2.29
After 12 months (*n* = 21)	36.90 ± 61.16	50.48 ± 7.93	34.90 ± 6.86	127.76 ± 40.22	50.24 ± 19.79	12.76 ± 2.10
After 24 months (*n* = 20)	35.90 ± 10.99	50.85 ± 14.59	32.05 ± 10.46	121.35 ± 59.03	46.20 ± 31.98	11.45 ± 4.48
*P* - value	0.001	0.195	0.063	0.003	0.004	0.004
Glucose						
Baseline (*n* = 22)	32.25 ± 5.88	46.88 ± 6.36	30.49 ± 6.14	103.95 ± 34.34	39.00 ± 22.22	11.53 ± 1.79
After 12 months (*n* = 21)	33.90 ± 5.08	47.24 ± 7.50	31.52 ± 6.61	106.43 ± 40.43	42.52 ± 23.15	11.33 ± 1.91
After 24 months (*n* = 18)	34.83 ± 5.45	49.22 ± 8.43	31.67 ± 7.47	118.39 ± 47.99	43.67 ± 27.06	11.33 ± 1.78
*P* - value	0.005	0.001	0.001	< 0.001	0.001	0.005
	LV systolic function		LV diastolic function			
	MPI	LVEF (%)	DT (ms)	Peak EDV (cm/s)	Septal EMV (ms)	*E*/*e*′
Icodextrin						
Baseline (*n* = 21)	0.37 ± 0.19	57.81 ± 10.03	169.81 ± 73.50	71.89 ± 34.66	5.43 ± 3.00	11.70 ± 6.36
After 12 months (*n* = 21)	0.43 ± 0.20	60.43 ± 7.29	214.24 ± 72.01	73.89 ± 27.94	6.05 ± 2.42	12.83 ± 6.60
After 24 months (*n* = 20)	0.32 ± 0.21	56.55 ± 21.88	193.35 ± 75.03	65.80 ± 33.68	5.51 ± 2.28	11.51 ± 9.60
*P* - value	0.195	0.068	0.081	0.150	0.035	0.104
Glucose						
Baseline (*n* = 22)	0.49 ± 0.57	63.40 ± 9.51	213.60 ± 85.21	70.67 ± 28.75	5.94 ± 1.58	12.38 ± 5.24
After 12 months (*n* = 21)	0.35 ± 0.18	60.67 ± 8.50	199.62 ± 79.76	66.52 ± 23.69	5.97 ± 3.11	10.46 ± 5.05
After 24 months (*n* = 18)	0.30 ± 0.19	64.50 ± 8.58	201.94 ± 62.55	68.25 ± 20.31	7.57 ± 3.42	9.08 ± 4.35
*P* - value	0.174	0.015	0.051	0.036	0.013	0.052

*P* value was estimated from Friedman test

*Abbreviation*: *DT* deceleration time

Table 4 presents the results of the generalized linear regression model for the association of clinical variables at baseline with changes in echocardiographic parameters at 24 months from baseline. No significant model was found in the present study. The multivariate models used to investigate changes in the four primary end points (MPI, LVEF, DT, and *E*/*e*′ ratio) are presented in Table 5. The primary end points show no significant association with the baseline values in both the ICO and GLU groups.

**Table 4:** Generalized linear regression analysis of the association between baseline clinical variables and changes in the echocardiographic parameters from baseline to 24 months

Dependent variable	RMSE	*R* ^2^	F	*P*
Cardiac structure				
LA diameter (mm)	7.43	0.88	0.75	0.716
LVEDD (mm)	15.00	0.89	0.82	0.680
LVESD (mm)	7.75	0.94	1.59	0.398
LVEDV (mm^3^)	62.12	0.88	0.70	0.745
LVESV (mm^3^)	14.64	0.97	3.44	0.168
IVS (mm)	2.74	0.94	1.61	0.392
LV systolic function				
MPI	0.25	0.98	4.37	0.125
LVEF (%)	22.33	0.87	0.65	0.778
LV diastolic function				
Deceleration time (ms)	54.74	0.96	2.42	0.256
Peak early diastolic velocity (cm/s)	44.19	0.87	0.66	0.768
Septal EM velocity (ms)	4.77	0.80	0.41	0.918
*E*/*e*′	12.68	0.79	0.38	0.932

All sociodemographic variables, PET variables, and icodextrin/glucose were included as covariates in the model

**Table 5:** Multivariate model analysis for the primary end points at 24 months

End Point	Parameter	Estimate	SE	*t*	*P*	n	*R* ^2^	Adjusted *R*^2^
MPI	Intercept	0.03	0.08	0.41	0.682	38	0.01	−0.05
	Baseline MPI	−0.02	0.07	−0.34	0.733			
	Icodextrin (vs. glucose)	0.31	0.05	5.60	0.000			
LVEF	Intercept	0.33	0.30	1.10	0.280	38	0.09	0.03
	Baseline LVEF	6.60	5.63	1.17	0.249			
	Icodextrin (vs. glucose)	37.01	18.20	2.03	0.050			
DT	Intercept	0.23	0.20	1.16	0.253	38	0.04	−0.01
	Baseline DT	3.92	22.80	0.17	0.864			
	Icodextrin (vs. glucose)	152.00	38.78	3.92	0.000			
*E*/*e*′	Intercept	0.32	0.27	1.18	0.248	35	0.06	0.00
	Baseline *E*/*e*′	−1.74	2.65	−0.66	0.517			
	Icodextrin (vs. glucose)	7.33	3.75	1.95	0.060			

## Discussion

In the present study, we evaluated the effects of the ICO solution dwell on cardiac structure and function of incident-APD patients by performing serial echocardiographic examinations for 2 years. Overall, most echocardiographic parameters of cardiac structure showed a significant change within the 2-year period in both ICO and GLU groups. Other echocardiographic parameters of LV systolic function and diastolic function showed varying significant changes in the ICO and GLU groups. Echocardiography has been recognized as a valuable and noninvasive tool to evaluate cardiac structure and function in CKD patients. It provides information not only on measurements of ventricular mass and volume status, either in systolic or diastolic phase, but also on geometry, ejection fraction, filling pressure, and valvular disease. The National Kidney Foundation’s Kidney Disease Outcomes Quality Initiative clinical practice guidelines for cardiovascular disease in dialysis patients recommends that echocardiography be performed in all patients (pediatric and adult) at the initiation of dialysis, once the patients have achieved dry weight (ideally within 1–3 months of initiation) and at 3-year intervals [10]. Several clinical studies have been conducted on the use of serial echocardiographies to evaluate cardiac structure and function in varying stages of CKD. In the subset patient analysis in a chronic renal insufficiency cohort study, results demonstrated that mean left ventricular mass index did not change from progression to advanced CKD to dialysis therapy initiation. However, ejection fraction declined during this transition period [11]. In the IDEAL (Initiating Dialysis Early and Late) trial, the investigators did not find any changes in echocardiographic parameters 12 months after dialysis initiation [3]. In the present study, we found that, in both groups, most of the echocardiographic parameters significantly differed in the 2-year study period. The above-mentioned observations imply that abnormalities in the cardiac structure and function of CKD patients resulted from uremic milieu, the progression of which could not be inhibited by dialysis initiation [12]. However, the echocardiographic results provide valuable information to clinicians in clinical practice. Based on this, a suitable therapeutic strategy combining dialysis regimen administration, drug adjustment, and lifestyle modification can be planned.

LAD has been hypothesized as an indicator of the integration of LV diastolic performance over time. Thus, left atrium (LA) volume provides a prospective view of diastolic dysfunction [13, 14]. Moreover, LA size is one of the most important echocardiographic risk predictors of LV hypertrophy. Prior studies have demonstrated the correlation between LA size and LV mass [14–16]. In the present study, results showed a LAD increment at 12 months in the ICO group and at 24 months in the GLU group. LAD is not reduced with either the ICO solution or the GLU solution over 24 months. Our result contradicts that of the study by Io et al., as they observed a reduction in LA size in PD patients after 24 months [14]. The exact explanation is not clear; however, the hemoglobin level in the Io study was indicative of a more severe anemia than that in the present study. We speculate that anemia management may be a contributing factor in LA size reduction in 24 months. However, the small sample size in both studies might have biased the results.

Congestive heart failure (CHF) is a common presenting symptom of cardiovascular disease in the dialysis population [17–21]. An earlier study demonstrated that the prevalence of CHF from the start of dialysis therapy and its subsequent annual incidence were high [18]. In the present study, CHF was not evidenced at baseline by indicators of LV systolic function (MPI and LVEF) and diastolic function (DT and *E*/*e*′). In the study by Wang, et al., elevated *E*/*e*′ ratio (> 15) was found in more than 50% of the prevalent PD patients [22]. *E*/*e*′ ratio was an independent risk factor for all-cause mortality and cardiovascular mortality [22]. The present study showed that the *E*/*e*′ ratio was progressively reduced after 24 months in the incident-APD patients, either in the ICO or GLU dwell. Nevertheless, other indicators of LV diastolic function in both groups did not change markedly at 24 months. The results indicate that ICO and GLU dwells in incident-APD patients maintain reasonable cardiac function over 24 months.

Residual renal function (RRF) not only provides a small solute clearance, but also maintains fluid control in PD patients. RRF supports the maintenance of cardiovascular health of PD patients [23]. RRF was significantly associated with LVH, independent of hypertension, anemia, and hypoalbuminemia [24]. In the present study, parameters of cardiac structure and cardiac function were not associated with RRF, indicated by weekly renal creatinine clearance in the generalized liner regression analysis. We expect future studies to clarify the association between RRF and whole cardiac function.

The ICO solution was characterized by a longer dwell time than that with the GLU solution. A meta-analysis of ICO solution in PD therapy demonstrated that ICO solution provided greater fluid removal and small solute clearance [6]. In the present study, we intended to investigate the association between changes in cardiac structure and function in ICO dwell in incident-APD patients. In the multivariate analysis, we did not find any advantage of the ICO dwell when compared with the GLU dwell in terms of the parameters of cardiac systolic (MPI and LVEF) and diastolic functions (DT and *E*/*e*′) for a 24-month period in incident-APD patients. Nevertheless, the ICO dwell maintains LV systolic and diastolic functions over 24 months. This result indicates that a long dwell time with ICO solution in daytime is not harmful to the cardiac function of APD patients.

Although the present study had a prospective randomized design, it still has some limitations. First, the participants had more favorable baseline characteristics, including age, blood pressure levels, and cardiac function. These characteristics may mask significant changes in echocardiographic parameters after PD initiation. Second, the sample size was relatively small and might have affected the statistical power of our analyses. Furthermore, cardiac structure and LV systolic function (LVEF) at baseline worsened more in the ICO solution group than in the GLU solution group, and might have masked the advantage of the ICO dwell over the GLU dwell in the period after APD initiation.

## Conclusions

The present study demonstrated that no major differences in cardiac structure and function were observed when comparing ICO and GLU solutions in incident-APD patients. Further studies, with larger sample sizes and longer study periods, are needed to evaluate the association between the ICO solution and changes in cardiac parameters by using echocardiographic examination.

## Abbreviations

*E*/*e*′: Peak early diastolic velocity/septal EM velocity
APD: Automated peritoneal dialysis
CHF: Congestive heart failure
CKD: Chronic kidney disease
EM: Early mitral annulus
HbA1C: Glycohemoglobin
hsCRP: High-resolution C-reactive protein
ICO: Icodextrin
iPTH: Intact parathyroid hormone
IVS: Interventricular septum
LA: Left atrium
LAD: Left atrial diameter
LV: Left ventricle
LVEDD: Left ventricular end-diastolic dimension
LVEDV: Left ventricular end-diastolic volume
LVEF: Left ventricular ejection fraction
LVESD: Left ventricular end-systolic dimension
LVESV: Left ventricular end-systolic volume
MPI: Myocardial performance index
PD: Peritoneal dialysis
PET: Peritoneal equilibration test
RRF: Residual renal function
